# Protocol for applying Tumor Treating Fields in mouse models of cancer using the inovivo system

**DOI:** 10.1016/j.xpro.2024.103535

**Published:** 2025-01-09

**Authors:** Travis J. Gates, Sewar Zbidat, Karina Deniz, Sanyukta Padmanabhan, Katherine Ladner, Akshat Sarkari, Xianda Zhao, Shiri Davidi, Roni Blatt, Shay Cahal, Martin Gabay, Mariell Sellevoll, Itai Tzchori, Yaara Porat, Adi Haber, Moshe Giladi, Subbaya Subramanian, Emil Lou

**Affiliations:** 1 Department of Pharmacology, University of Minnesota Medical School, Minneapolis, MN, USA; 2 Novocure Ltd., Haifa, Israel; 3 Department of Medicine, Division of Hematology, Oncology, and Transplantation, University of Minnesota Medical School, Minneapolis, MN, USA; 4 Department of Surgery, University of Minnesota Medical School, Minneapolis, MN, USA; 5 Masonic Cancer Center, University of Minnesota Medical School, Minneapolis, MN, USA; 6 Center for Immunology, University of Minnesota Medical School, Minneapolis, MN, USA

**Keywords:** cell biology, cancer, model organisms

## Abstract

Tumor Treating Fields (TTFields) are electric fields clinically approved for cancer treatment, delivered via arrays attached to the patient’s skin. Here, we present a protocol for applying TTFields to torso orthotopic and subcutaneous mouse tumor models using the inovivo system. We guide users on proper system component connections, study protocol design, mouse fur depilation, array application, and treatment condition adjustment and monitoring. The inovivo system allows for the concurrent application of TTFields with standard cancer therapies.

For complete details on the use and execution of this protocol, please refer to Barsheshet et al.^1^

## Highlights

• Instructions for setup of the inovivo system
• Steps for assembly of TTFields and sham arrays to mice torsos
• Guidance on monitoring an inovivo experiment

## Abstract (editor highlights)

Publisher’s note: Undertaking any experimental protocol requires adherence to local institutional guidelines for laboratory safety and ethics.

## Graphical abstract

**Figure:**
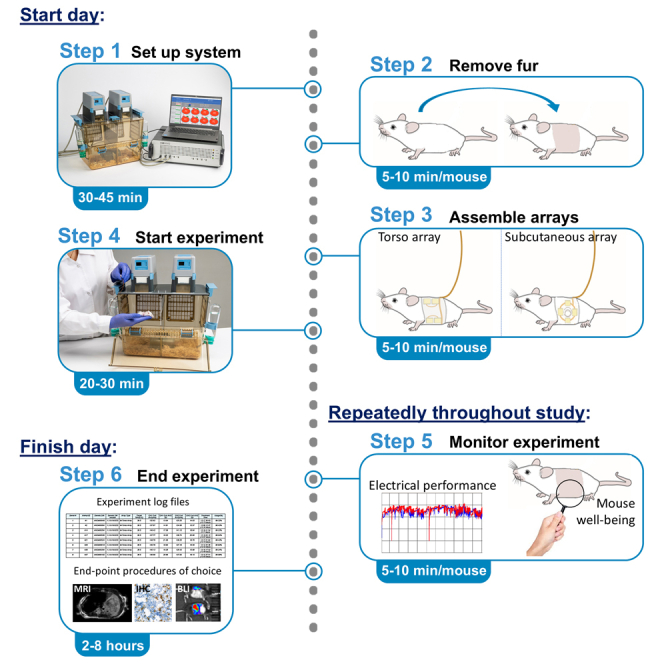

## Before you begin

TTFields therapy is FDA approved for treatment of glioblastoma and unresectable pleural mesothelioma and is under clinical investigations for the treatment of other types of solid tumors.^2^ TTFields are delivered non-invasively, using a portable field generator and two pairs of intermittently operating arrays attached to the patient’s skin surrounding the tumor region.^2^ Effective treatment with TTFields requires continuous application (clinical recommendations are for usage of at least 18 h/day, i.e., above 75%), necessitating optimal long-lasting array contact with the patient’s skin.^3^

TTFields delivery to mice requires multi-wired conductors coupled to array electrodes placed on their torsos throughout the entire treatment period. As such, the treatment limits animal movement and requires individual housing to prevent wire entanglements, altogether imposing stress on the mice. Previously, *in vivo* studies utilized an adapted human system,^4^ not ideally designed to address these challenges, compromising the possibility of performing studies in animal models. The new inovivo system is a dedicated system for treating mice with TTFields, aiming to overcome the challenges by using arrays and cages with improved designs.

The inovivo system can deliver TTFields to torso orthotopic and subcutaneous (SC) tumor-bearing mice, with or without concurrent application of other cancer treatments, such as chemotherapy, immunotherapy, or irradiation. Importantly, the application of TTFields generates local heating underneath the arrays. To take into account this heating and the stress imposed on the mice by the presence of arrays on their torsos, sham arrays generating only heat are used as control conditions for TTFields.

The protocol below has been performed with the LL/2-Luc2 mouse lung cancer cell line inoculated orthotopically to the lung of 10–12 week old male C57BL/6J mice^1^; and with the mouse colorectal cancer cell line CT26 inoculated SC to 6–8 week old female BALB/C mice. However, it may be performed with other cell lines and mouse models of the investigator’s choice, in torso orthotopic or SC format.

### Institutional permissions

This protocol describes experiments on live animals and must be performed in accordance with local and/or national guidelines and must follow appropriate regulations. The experiments described in this protocol were approved by the Novocure institutional animal care and use committee (IACUC) and the Israeli National Committee Council for Experiments on Animal Subjects (approval numbers IL-2111-104-4 and IL-2202-104-4) or by the IACUC at the University of Minnesota protocol (#2107-39273A).

### Design your experiment

The inovivo system supports the treatment of both torso orthotopic and SC (compatible with right flank, but if needed may be adjusted to left flank) tumors. The investigator chooses the specific mouse model and tumor cells for inoculation. However, the cages do not support specific-pathogen-free (SPF) conditions and are hence not compatible with immunodeficient models (this may however be made possible by maintaining specific conditions; please refer to the limitations section).

When designing the experiment, one may consider implementing scheduled treatment breaks into the study. Such breaks may be included to provide the mice time for rest and recovery without the arrays. Scheduled imaging procedures performed throughout treatment may be exploited for this purpose, as the imaging process requires removal of the arrays. Please keep in mind that continuous treatment is pivotal for treatment efficacy, and hence breaks should be kept as short as possible.

### Identify the correct TTFields frequency for the tumor type you are treating

TTFields treatment frequency is tumor-type-specific (determined empirically within the range of 100–500 kHz) and should be identified before starting an *in vivo* study. Refer to the scientific literature to find the accurate frequency for the tumor type you are using.^5^^,^^6^ If such has not been published, *in vitro* frequency scans should be performed to identify the optimal frequency for that tumor type.^7^

### Get to know the inovivo system components

The inovivo system consists of arrays, generators, swivels, cages, software, and accessories.

**Figure 1:**
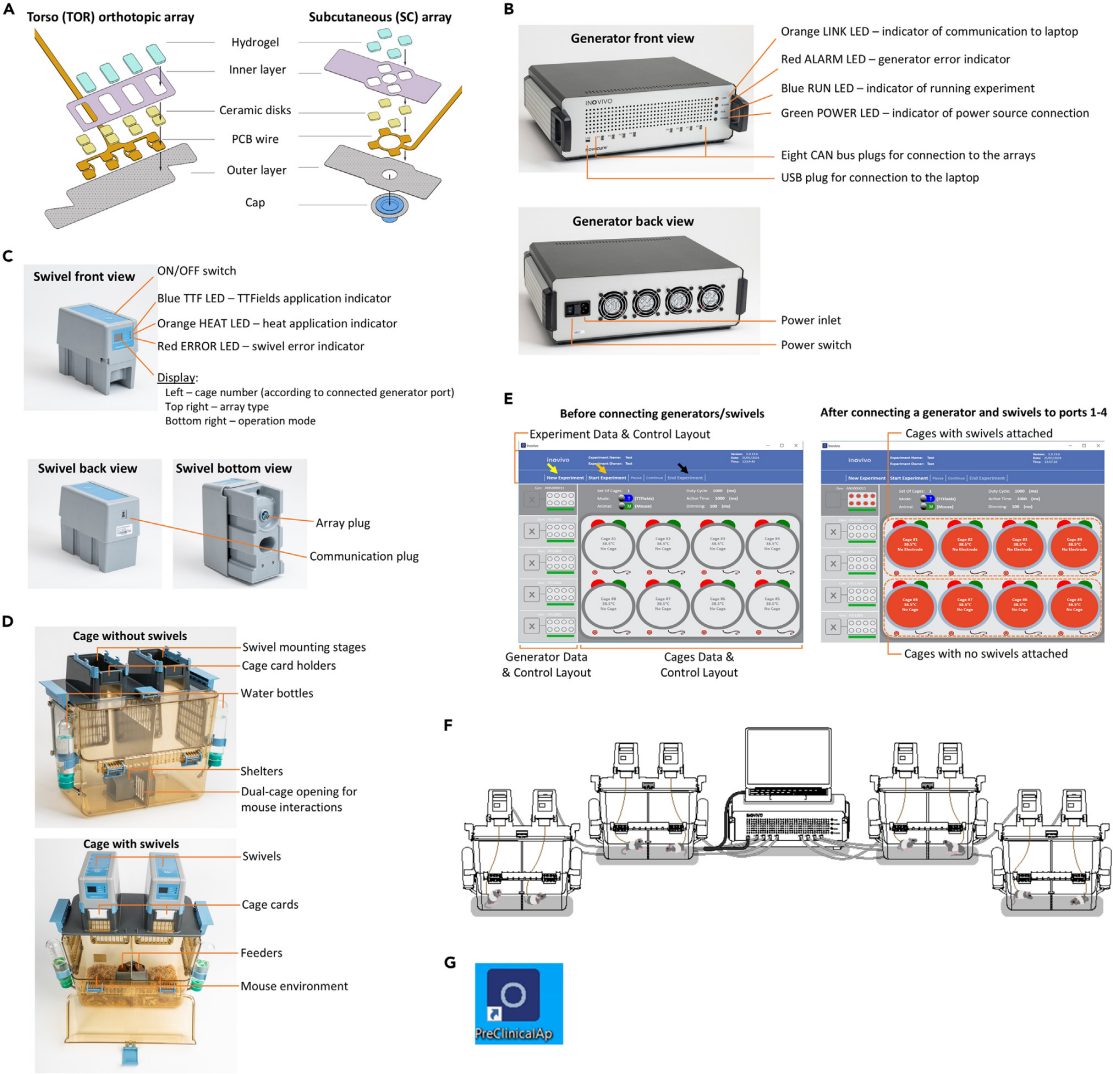
Get to know the inovivo system components and set up the system Arrays (A). Generators (B). Swivels (C, for a better view of the display refer to Figure 6D). Cages (D). Software screenshots before and after connecting 1 of 5 possible generators and 4 of 8 possible mice/swivels (4 dual cages), new/start/end experiment indicated with yellow/orange/black arrows, respectively (E). Scheme of a fully connected inovivo system (F). The desktop software icon (G).

**Arrays** (Figure 1A) – Arrays are available for TTFields or sham (heat) treatment for torso orthotopic and SC mouse tumor models. The arrays consist of two pairs of ceramic disks (referred to as channel 1 and channel 2), to be placed orthogonally around the tumor region, that operate intermittently. The TTFields arrays contain temperature-measuring components (referred to as P1 and P2) located on the rear side of the ceramic disks, incorporated to prevent skin burns due to overheating. The sham arrays include built-in components to generate heat equivalent to that produced by the TTFields arrays. The ceramic disks are connected to a single flat, flexible, lightweight printed circuit board (PCB) wire. The arrays consist of two layers: (1) a thin, flexible inner-layer, which allows improved yet gentle adherence to the mouse skin; (2) a soft, lightweight outer-layer to secure arrays to the skin. A medical gel covers the ceramic disks to permit electrical conductivity.

**Generators** (Figure 1B) – Each generator provides treatment for up to 8 mice. Two generators are required for performing an inovivo experiment, one set to apply TTFields (at a selected frequency in the range of 100–500 kHz), and the other to generate heat (sham treatment). The thermistor readings from the TTFields arrays are transferred to the generator, which adjusts the output voltage separately for each mouse as required to prevent overheating. The generators are supplied with USB cables for connection to the laptop. Power cords are not supplied.

**Swivels** (Figure 1C) – The swivel is a motor device that serves as the point of connection of the array to the generator. The swivel constantly senses animal movement and rotates accordingly to prevent cable coiling that would interfere with treatment application. The swivels are supplied with CAN bus communication cables for connection to the generators.

**Cages** (Figure 1D) – The inovivo cages are designed to house two mice, each of them individually, with an opening enabling interaction. The individual housing prevents entanglement of the wires from different mice and the possibility of one mouse damaging the array of the other mouse. The opening of the dual cage allows social interaction between the two mice, hence reducing their stress during treatment. Each mouse environment also contains a feeder, a water bottle, and a shelter.

**Software** (Figure 1E) – The inovivo software runs on the dedicated supplied laptop. The software displays which generators and related cages are available (Generator Data & Control Layout) and which animals/swivels are connected (Cages Data & Control Layout). It allows experimental control and parameters setting (e.g., TTFields frequency) for each mouse. It also monitors experimental parameters in real-time and records them for later analysis. The software will send alerts when operational faults occur.

**Accessories package** – The package contains an adhesive strip roll needed to secure the arrays to the mice, cage cards, filters, and an autoclave bottle basket.

### Set up the inovivo system

**Timing: 3–4 h**

Before first use, the inovivo cages should be assembled, washed, autoclaved, and two swivels mounted on top of each cage. The generators should be connected to the swivels and to the laptops with the inovivo software (Figure 1F). If during system assembly there is any deviation from the description below, please refer to troubleshooting 1.

1. Assemble the inovivo cages according to the instructions in the inovivo user manual supplied with the system and position the cages in place.

**CRITICAL:** Cages should be cleaned and autoclaved before each experiment. Swivels are not autoclavable and should be cleaned with wipes.

**CRITICAL:** Place cages such that they are not located directly in front of ventilation outlets.

2. Position the laptops and generators in place.

***Note:*** The laptops and generators should be close to the cages and to one another to allow cable connection of all system components. To accommodate a generator, a laptop and four cages (i.e. one inovivo systems), a minimal bench area of 250 cm × 50 cm is needed, with an overhead space of 45 cm.

3. Place the swivels on top of the cages in their designated positions.

**CRITICAL:** Only attach swivels to cages already in place to avoid damage to these sensitive components when moving around cages.

4. Connect each generator to a laptop through the provided USB cables.
5. Connect the generators and laptops to a power source using standard power cables.
6. Connect the generators to the connection plugs at the back of the swivels through the provided CAN bus communication cables.
7. Turn on the laptops.
8. Open the inovivo software by double-clicking the designated icon on the computer desktop (Figure 1G).
9. Turn on the generators by pressing the power switch on the back of each generator.

***Note:*** Initially, all 4 generator LEDs will turn on, accompanied by a beeping sound (Figure 1B). Then the beeping will stop, and all LEDs will turn off, except for the green power LED. Next, the orange LED indicating communication with the software will turn on.

10. Check the software to see an indication of generator connection – the circles denoting the cages will turn from empty to filled red on both the Generator Data & Control Layout and the Cages Data & Control Layout (Figure 1E).
11. Check the displays on the swivels (Figure 1C) to verify each is showing the number of the generator port to which it is connected.
12. Check the software to see indication of swivel connection – the cages in the Cages Data & Control Layouts (Figure 1E) will display “no electrode” (instead of “no cage”).
13. Turn off the generators, remove the swivels, and store all system components in a safe and dust free place until your experiment starts.

### Generate tumor-bearing mice

**Timing: 2–8 h**

14. Acquire the required mice (according to your experimental design) and maintain them in the animal facility under standard conditions.

**CRITICAL:** For good arrays attachment, mice should weigh at least 20 g when starting an inovivo experiment.

15. Prepare tumor cells and inoculate them to the mice according to your model and protocol of choice.

**CRITICAL:** SC tumors should be injected higher than the common injection site to allow array application with minimal interference to the mouse back limbs. The injection site should be on the right side of the torso, at the level of the last rib or just caudal to it (head-tail axis) and at the midpoint between the back and the abdomen (dorso-ventral axis). It is recommended to use a SC array as a template to identify the appropriate injection site.

**CRITICAL:** Before you continue to assemble the arrays, make sure the mice do not have open wounds. Arrays may only be placed on healthy skin. If an invasive procedure was performed, wait until sutures are fully healed before continuing to array assembly.

16. Allow a few days (according to your experimental design) for tumors to grow before starting treatment.

## Key resources table

**Table:** 

REAGENT or RESOURCE	SOURCE	IDENTIFIER
**Experimental models: Cell lines**		

LL/2-Luc2	ATCC	CRL-1604
CT26	ATCC	CRL-2638

**Experimental models: Organisms/strains**		

C57BL/6J mice, wild-type, 6–8 weeks, 20–24 gr, male	Envigo CRS, Israel	N/A
BALB/C, wild-type, 6–8 weeks, 20–24 gr, female	The Jackson Laboratory	Strain #000651

**Chemicals, peptides, and recombinant proteins**		

DietGel 76A	ClearH_2_O	72-07-5022X
HydroGel	ClearH_2_O	70-01-5022
Medical adhesive remover spray	Silmed	SIL-079
Synthomycine 5%	Vetmarket	134001
Nair	iHerb	NAI-28001
Sterile NaCl solution	Vetmarket	185256
IODO-VIT solution	Vetmarket	147140
Ringer’s lactate solution	B. BRAUN	3629619
7093 Teklad shredded aspen bedding	Inotiv	7093 Teklad

**Software and algorithms**		

inovivo software	Novocure	N/A

**Other**		

inovivo arrays	Novocure	N/A
inovivo generators	Novocure	N/A
inovivo swivels	Novocure	N/A
inovivo cages	Novocure	N/A
Laptop with inovivo software	Novocure	N/A
inovivo USB cables	Novocure	N/A
inovivo CAN bus cables	Novocure	N/A
Power source cables	N/A	N/A
inovivo SC array caps	Novocure	N/A
inovivo roll of adhesive strips	Novocure	N/A

## Step-by-step method details

Please note that the required time for completion of some protocol steps depends on the number of mice included in the study, and therefore for such steps timing is given per mouse. When a few researchers conduct the study together, work can be done in parallel on several mice. The recommendation would be to have each researcher in charge of a specific part of the protocol, so that the mice move between researchers, rather than one researcher working with a specific mouse from start to end.

### Set experimental conditions on the inovivo system

**Timing: 30–45 min**

Before starting mice sedation, the cages need to be prepared and the system needs to be installed, turned on, and verified for proper operation. When the system is turned on, experimental parameters may be set.

Required equipment: inovivo system components (cages, swivels, generators, laptops, cables, water bottles), sawdust (7093 Teklad Shredded Aspen Bedding, 7099P TEK-Fresh Bedding, or any other bedding of similar dimensions), food (DietGel 76A or any other supplementary food with a high moisture content of 60%–75% and a high caloric content, at least 1 kcal per 1 g of food).

***Note:*** Non-wetting water gel may be used for animal hydration (HydroGel) if animal water intake is insufficient.

1. A few days before the study begins, prepare the inovivo cages with an environment supporting animal necessities (fill the water bottles, spread sawdust, and provide food). Transfer the mice into the cages.

***Note:*** Introducing an acclimation period of the mice to the inovivo cage prior to study initiation reduces animal stress. During the study, the use of a soft nutritionally fortified dietary supplement is suggested, which also requires prior adaptation.

2. When ready to begin the study, take the remaining system components out of storage and position them in place.
3. Follow steps 3–12 of the set up the inovivo system section to turn on the system and verify it is running correctly.

***Note:*** If there is any deviation from the description, please refer to troubleshooting 1.

**Figure 2:**
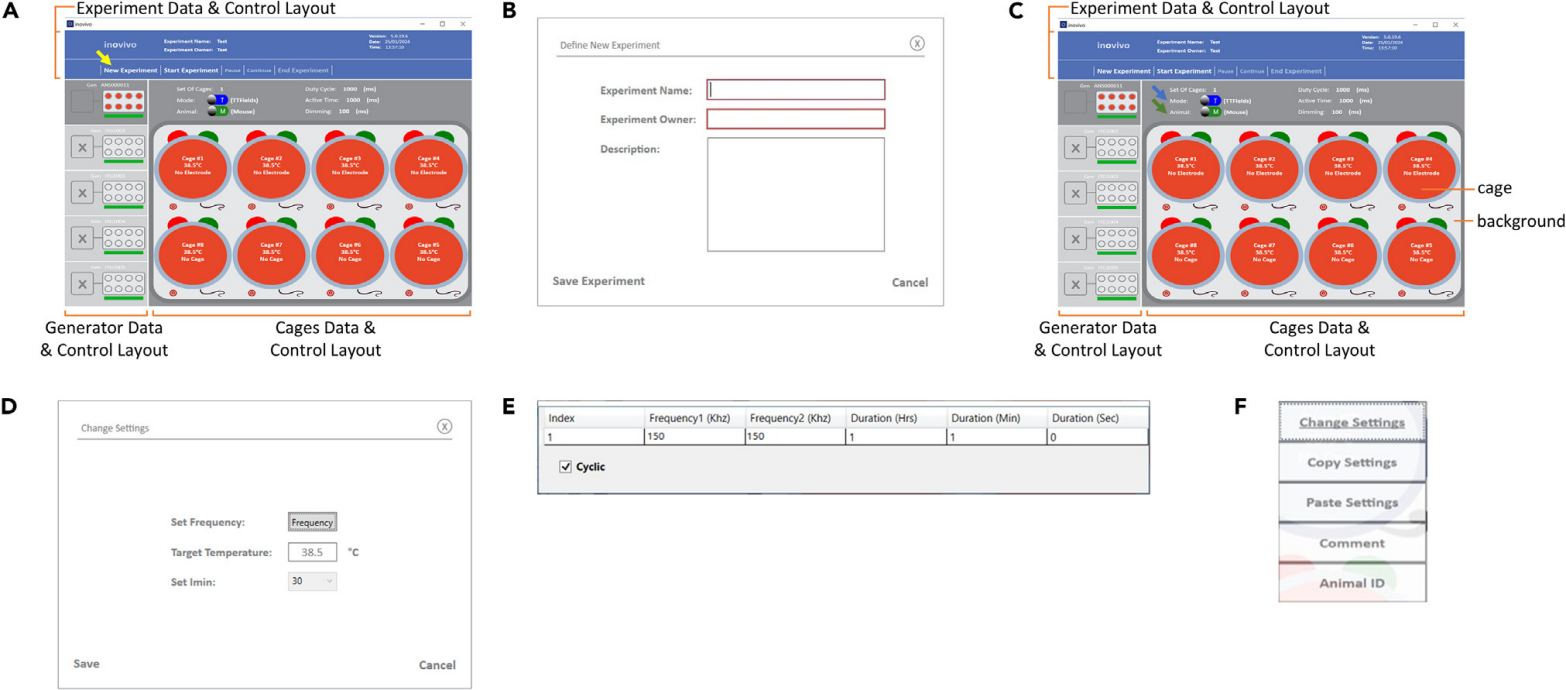
Turn on the inovivo system and set experimental conditions Use the inovivo software to set the treatment parameters: Press New Experiment (indicated with a yellow arrow) (A); In the opened window, insert experiment name, owner, and description (B); Set generator experimental parameters, selecting the animal type (mouse/rat, indicated with a green arrow) and experiment mode (TTFields/heat, indicated with a blue arrow) for each generator (C). Set first cage experimental parameters, selecting the frequency and target temperature (D and E); Copy the parameters to the other cages (F).

4. Start a new experiment. Within the software on each computer:
  a. Press the New Experiment button in the Experiment Data & Control Layout (Figure 2A, indicated with a yellow arrow).
  b. In the pop-up window, type experiment name and owner; and possibly experiment description (Figure 2B).
  c. Press the Save Experiment button (Figure 2B).
5. Set experimental parameters for each generator:
  a. Select the generator in the Generator Data & Control Layout.
  b. Verify in the Cages Data & Control Layout (Figure 2C, indicated with a green arrow) that the animal type is set to M (mouse) or correct if needed.
  c. Select in the Cages Data & Control Layout (Figure 2C, indicated with a blue arrow) the experiment mode for each generator, T (TTFields) or H (heat).
  d. Set treatment parameters for the first mouse:
    i. Right-click on a cage in the Cages Data & Control Layout (Figure 2C, indicated with “cage”) to open the parameters adjustment pop-up window (Figure 2D).
    ii. For the TTFields generator – set the treatment frequency (Figure 2E).
      ***Note:*** Treatment frequency should be defined beforehand (see identify the correct TTFields frequency for the tumor type you are treating section).
    iii. For both TTFields and heat generators – set the target temperature at 38.5°C.
  e. Copy the parameters to the other cages:
    i. Right-click on a cage in the Cages Data & Control Layout (Figure 2C, indicated with “cage”) from which the parameters should be copied.
    ii. Select Copy Settings from the pop-up window (Figure 2F).
    iii. Right-click on a background in the Cages Data & Control Layout (Figure 2C, indicated with “background”).
      ***Note:*** Selecting the background will paste the copied setting to all cages. You may alternatively select a specific cage to which the settings should be copied.
    iv. Select Paste Settings from the pop-up window (Figure 2F).

### Prepare the mice by fur removal from the torsos

**Timing: 10–15 min per mouse**

Effective treatment with TTFields requires good and continuous contact of the arrays with the skin, so mice must be depilated before arrays are applied.

**Figure 3:**
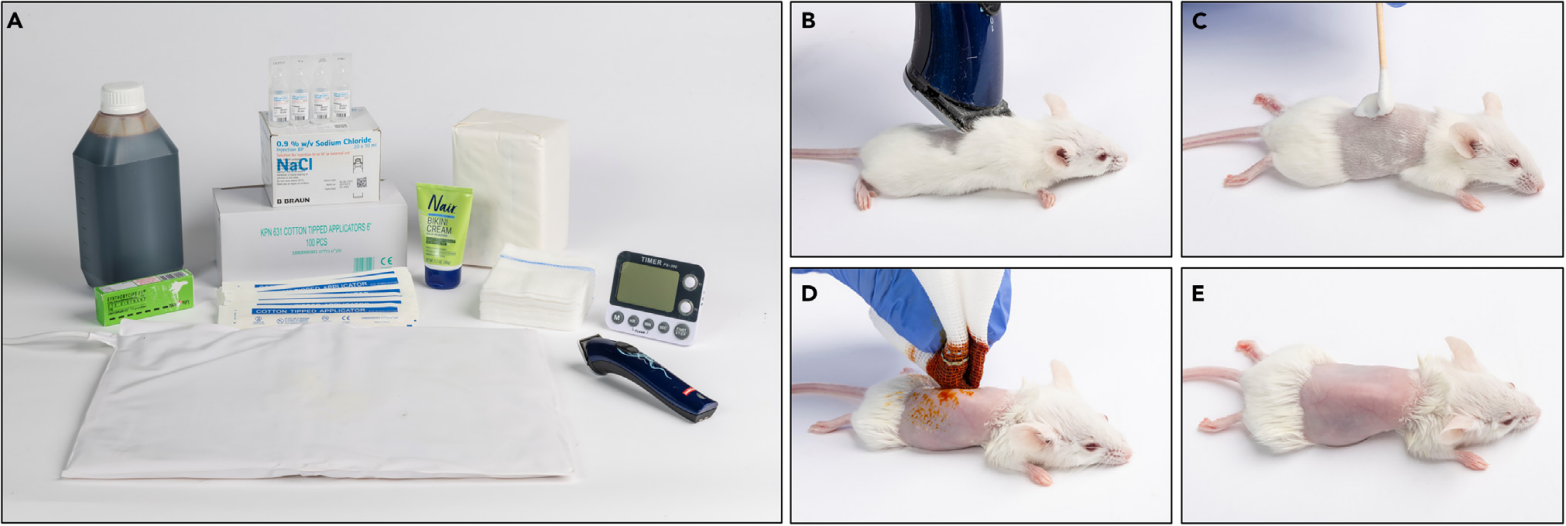
Remove fur from the mice torsos The required materials and equipment: disinfection agent, eye lubricant product, sterile NaCl solution, cotton applicators, hair depilation cream, gauze pad, stopwatch, heating pad, and trimming machine (A), and the procedure steps: Trim the fur from the torso of the mouse (B); Add hair depilation cream to the shaved regions (C); Apply disinfection agent to the depilated area (D); Allow mouse skin to dry completely (E).

Required equipment (Figure 3A): trimming machine, heating pad, eye lubricant product (Synthomycine 5%, or any other lubricating ophthalmic ointment), cotton applicators, stopwatch, hair depilation cream (Nair, Veet, or any other depilating cream), gauze pad, sterile NaCl solution, disinfection agent (IODO-VIT solution, Septal scrub, or any other disinfection agent), device for mouse marking (not shown), and anesthetics of choice (not shown).

6. Turn on the heating pad.
7. Mark the mouse for identification.
8. Weigh the mouse.

***Note:*** This will serve as a first optional “without array” initial mouse weight to define possible weight loss throughout the study.

9. Sedate the mouse utilizing the anesthetic(s) of choice.

***Note:*** Initial fur removal and array placement require time and handling. Choose an anesthetic protocol that allows for 30–40 min of sedation without the need for constant anesthetic supply.

10. Place the mouse on the heating pad.
11. Apply the eye lubricant product to the mouse’s eyes using a cotton applicator.
12. Trim the fur from the torso of the mouse using a suitable trimming machine (Figure 3B).

**CRITICAL:** Trim with care to avoid wounds and cuts, arrays may only be placed on healthy skin.

13. Add hair depilation cream to the shaved regions (Figure 3C).
14. Wait 1 min.

**CRITICAL:** Do not leave on depilating cream for more than one minute, this may hurt the skin.

15. Gently wipe off the depilating cream with a gauze pad soaked with warm sterilized saline.

***Note:*** Be very gentle to avoid damaging the skin.

16. Verify that the animal’s torso is clean from fur, and if not repeat stages 12–14.

***Note:*** To avoid damaging the skin, do not use hair depilating cream for more than 2 times per mouse. Limit re-application of depilation cream only to the regions with residual fur.

17. Apply the disinfection agent to the depilated area using a gauze pad or wipes (Figure 3D).
18. Wait 3 min.
19. Gently wipe off the disinfection agent with a gauze pad soaked with warm sterilized saline.

***Note:*** Be very gentle to avoid damaging the skin.

20. Allow mouse skin to dry completely before moving to the next step (Figure 3E).

### Assemble the arrays to the mice torsos

**Timing: 5–10 min per mouse**

Arrays may deliver TTFields or sham heat (equivalent to that generated by the TTFields arrays). The two types of arrays are identically assembled on the mouse torsos. There are arrays for treating torso orthotopic or SC tumors, which require somewhat different application procedures.

**Figure 4:**
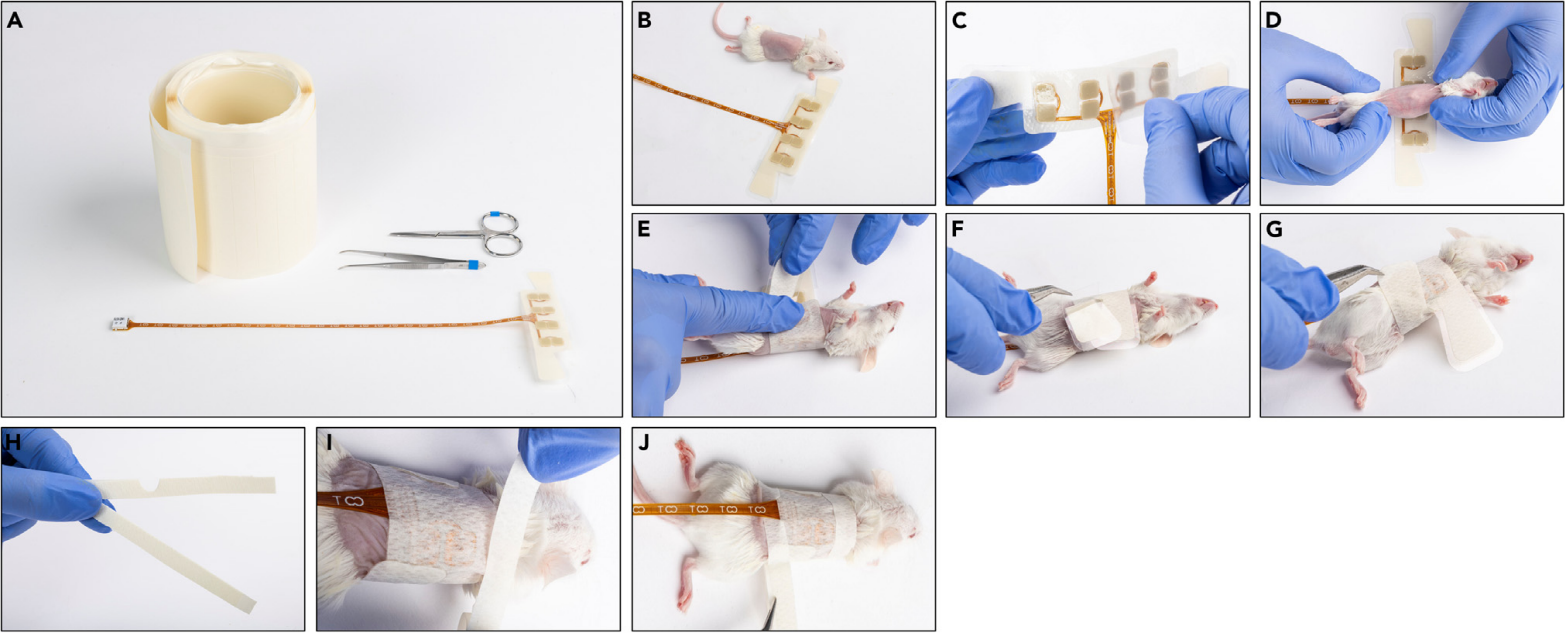
Assemble the torso orthotopic inovivo arrays to the mice torsos The required materials and equipment: roll of adhesive strips, tweezers, scissors, and array (A), and the procedure steps: Spread the array on the surface and verify the ceramic disks are intact (B); Peel the center liner (C); Place the mouse with its back on the center of the array with the tail pointing at the direction of the PCB wire (D); Attach the side of the array adhesive tape to the mouse skin (E); Peel the side liner from the array left wing (F); Wrap the wing around the animal torso, and attach it to array exterior (G); Repeat for the adhesive right wing; Prepare 2 adhesive strips (H); Attach the strip with no opening to the upper side of the array (I); Attach the strip with an opening to the lower side of the array (J).

**Figure 5:**
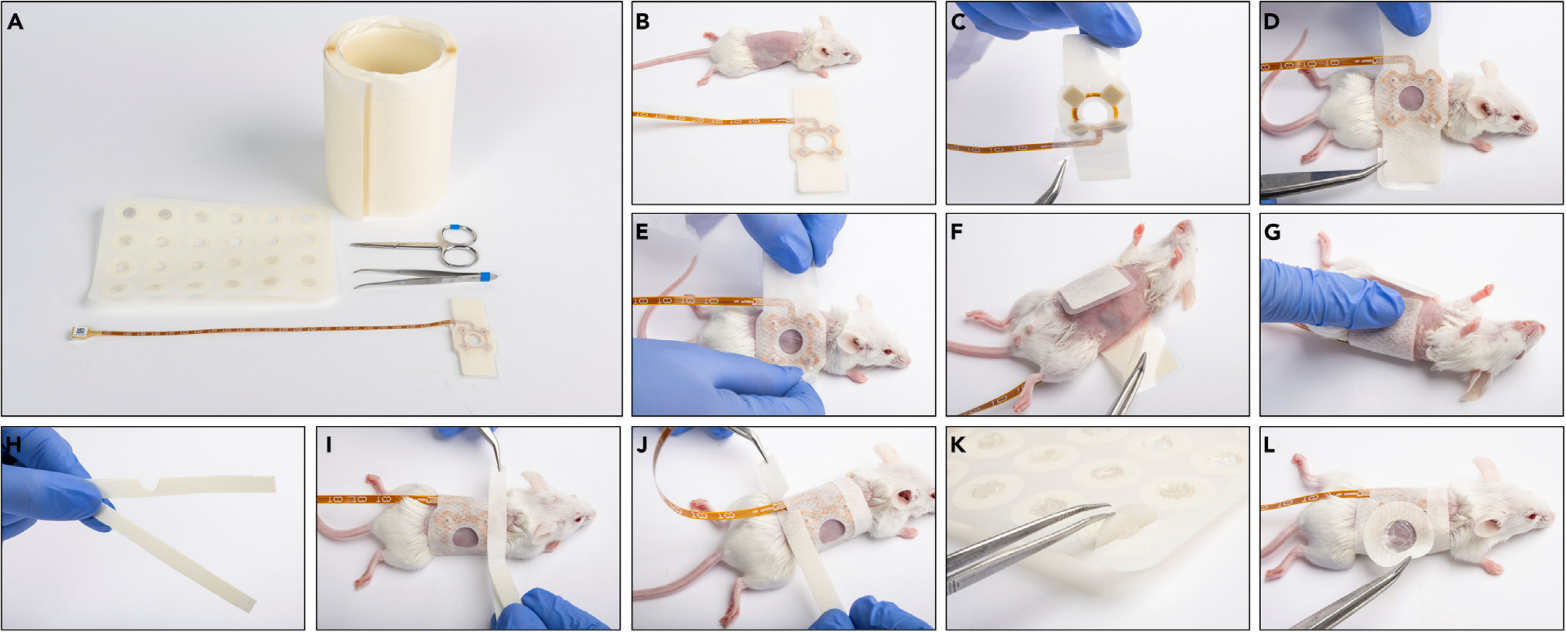
Assemble the subcutaneous inovivo arrays to the mice torsos The required materials and equipment: array caps, roll of adhesive strips, scissors, tweezers, and array (A), and the procedure steps: Spread the array on the surface and verify the ceramic disks are intact (B); Peel the center liner (C); Place the mouse on its stomach and place the array such that the tumor is located in the center of the circular opening in the array, with the PCB wire pointing in the same direction as the animal tail (D); Attach the sides of the array adhesive tape to the mouse skin (E); Peel the side liner from the array left wing (F); Wrap the wing around the animal torso, and attach it to array exterior (G); Repeat for the adhesive right wing; Prepare 2 adhesive strips (H); Attach the strip with no opening to the upper side of the array (I); Attach the strip with an opening to the lower side of the array (J); Remove a protective cap from the roll (K); Attach the cap on the tumor (L).

Required equipment: arrays, scissors, tweezers, roll of adhesive strips, and caps for SC arrays (Figures 4A and 5A for torso orthotopic and SC arrays, respectively).

21. Verify that the array package is intact, unpack the array, and verify the ceramic disks are intact (Figures 4B and 5B).

***Note:*** Once the array package is opened, the array should be used immediately to maintain sterility.

22. Hold the base of the array in both hands, and peel off the center liner (Figures 4C and 5C).
23. For orthotopic arrays follow (a), for SC arrays follow (b):
  ***Note:*** Isoflurane inhalation may be considered during the array assembly procedure.
  ***Note:*** Verify that the adhesive is not placed on fur.
  a. Orthotopic array: Place the array on the heating pad with the ceramic disks facing up and lay the mouse with its back on the array and tail pointing in the same direction as the array cable (Figure 4D).
    ***Note:*** Specific array location on the mouse torso depends on tumor location. For example, place the array as cranial as possible for the treatment of the lung and as caudal as possible for the treatment of the ovary. However, be mindful not to locate the arrays too close to the mouse’s limbs, as to avoid interference with the animal’s ability to move freely.
  b. SC array: Place the mouse on its stomach and place the array such that the tumor is located in the center of the circular opening in the array, with the array cable pointing in the same direction as the animal tail (Figure 5D).
24. Attach the side of the array adhesive tape to the mouse skin (Figures 4E and 5E).
25. Peel the liner from one of the array’s wings (Figures 4F and 5F), wrap it around the animal torso, and attach it to the array exterior (Figures 4G and 5G for torso orthotopic and SC arrays, respectively). Then, do the same for the other wing.

**CRITICAL:** The wrapping should be tight enough to ensure proper contact of the electrodes with the skin, but not too tight so that it would be uncomfortable for the mouse. Make sure the array is well attached.

26. Remove an adhesive strip from the roll and cut it into two longitudinal equal parts. Cut an opening in the middle of one of the strips (Figures 4H and 5H).
27. Attach the strip with no opening to the upper side of the array so that it adheres to both the skin and the array (Figures 4I and 5I).
28. Attach the strip with an opening to the lower side of the array so that it adheres to both the skin and the array with the opening below the PCB wire (Figures 4J and 5J).
29. SC array: install a protective cap on top of the tumor – peel the cap from the roll (Figure 5K) and attach it to the array exterior such that it covers the tumor (Figure 5L).
30. Weigh the animal with the attached arrays.

***Note:*** This will serve as a second optional “with array” initial mouse weight to define possible weight loss throughout the study.

31. Keep the mouse heated until full recovery.

***Note:*** When using alpha 2 agonists for animal sedation (e.g., xylazine, medetomidine) it is recommended to administer an antagonist drug (e.g., atipamezole, yohimbine) at this stage to promote faster recovery.

### Connect the mice to the inovivo system and start the experiment

**Timing: 20–30 min**

After the arrays have been placed, the mice need to be connected to the system, and the software used to commence the experiment.

**Figure 6:**
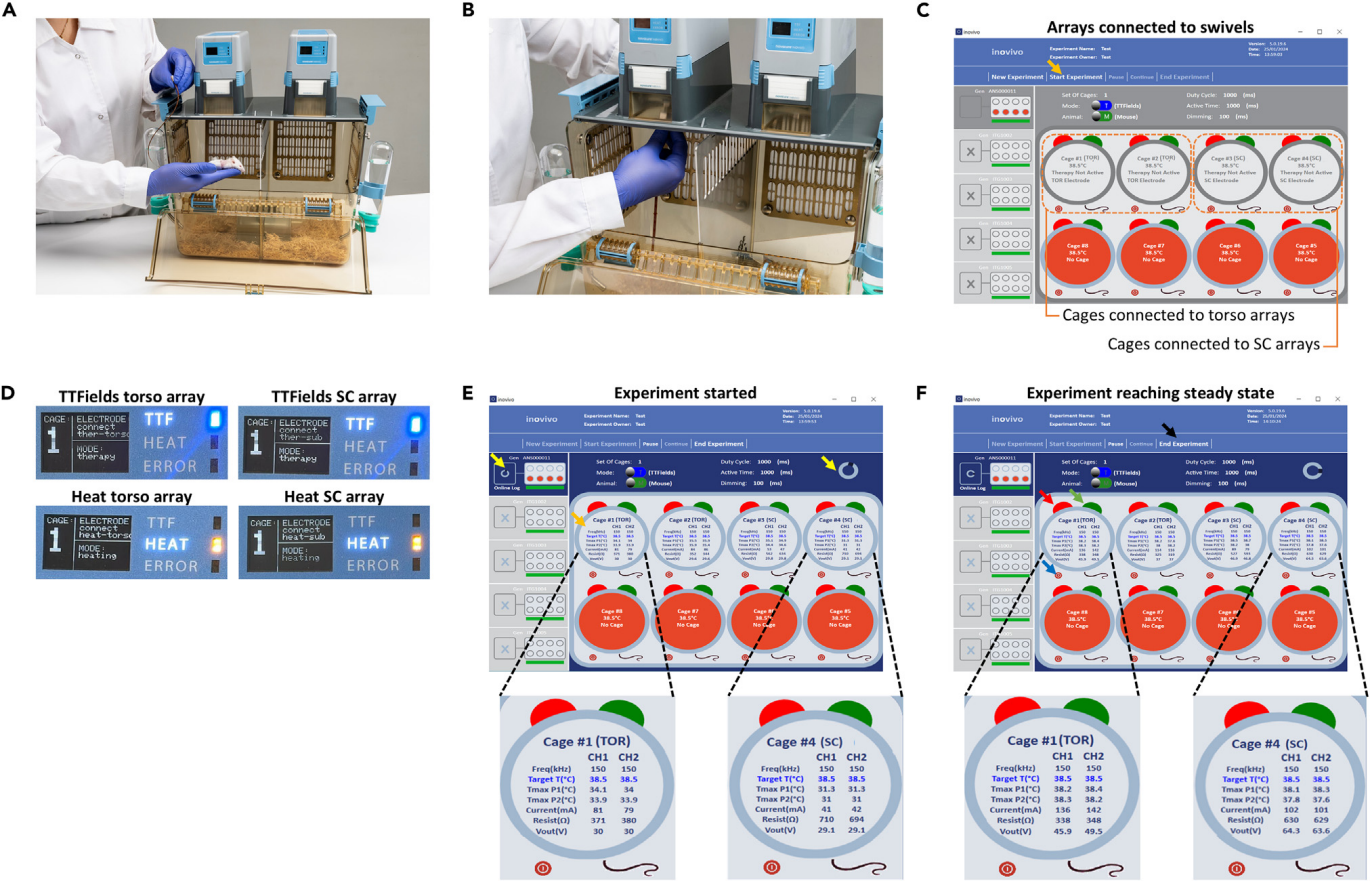
Connect the mice to the inovivo system and start the experiment Place the mouse in the cage once it is awake (A); Gently connect the array connector to the array plug on the bottom of the swivel (B); Verify swivel-array and generator-swivel connections in the software and on the swivel display (C and D); Examine the software interface to verify the experiment has started by the appearance of two “turning wheels” (indicated with yellow arrows) and measured parameters for the cages (indicated with an orange arrow for cage 1 as an example) (E), and that it has reached a steady state (F). For experimental control there is a master End Experiment button (indicated with a black arrow), and Pause, Resume, and End Experiment buttons per each cage (indicated with a red, green, and blue arrows, respectively, for cage 1 as an example) (F).

32. Once the mouse is awake, hold the array cable in one hand and use the other hand to place the mouse in the cage (Figure 6A).
33. Gently connect the array connector to the array plug on the bottom of the swivel (Figure 6B).

**CRITICAL:** Apply minimal pressure on the plug to prevent damage to this sensitive component, while verifying the connector is fully inserted into the plug.

**CRITICAL:** Make sure that each array is connected to the generator set to the appropriate mode (TTFields arrays to TTFields generator, heat arrays to heat generator).

34. Verify the connection of the swivel to the array and to the generator by checking the following, and if not refer to troubleshooting 2.
  a. The “cage” within the software Cages Data & Control Layout is showing the label "Therapy Not Active" and the type of the connected array ("TOR" or “SC”) (Figure 6C).
  b. The swivel display screen shows the type of the connected array (“ther-torso”, “heat-torso”, “ther-sub”, or “heat-sub”) and the operation mode (therapy or heating, as opposed to Idle), and the LED indicating TTFields or heat is illuminated (Figure 6D for the four available possibilities).

***Optional:*** Write experimental details on the cage card (supplied in the accessories package) and insert the card into its place in the cage card holder (Figure 1D).

35. Press Start Experiment in the software to begin the experiment (Figure 6C, indicated with an orange arrow).

***Note:*** The generators will turn on the blue RUN LED (Figure 1B). TTFields swivels will turn on the blue TTF LEDs, and heat swivels will turn on the orange HEAT LEDs (Figure 6D). Within the software, two “turning wheels” will appear, one in the Generator Data & Control Layout and one in the Cages Data & Control Layout, and the “cages” will show the measured temperature and electrical parameters for each channel (Figure 6E, indicated with a yellow and orange arrow, respectively).

***Note:*** If there is any deviation from the description, please refer to troubleshooting 2.

36. Check that electrical parameters have reached the desired values (Figure 6F; Table 1). Reaching a steady state may take up to 40 min. If the steady state is not reached, please refer to troubleshooting 3.

***Optional:*** Experimental comments and animal IDs may be added at any time via the software by right-clicking on the cage caption and following the on-screen instructions.

**Table 1:** Desired electrical parameters range for torso orthotopic (TOR) and SC therapy arrays

Parameter	TOR arrays	SC arrays
**Current (mA)**	100–150	70–100
**Resistance (Ω)**	200–400	400–700
**Voltage**_**out**_**(V)**	30–40	40–50

### Monitor mice and arrays throughout the experiment

**Timing: 10–20 min per mouse**

During the experiment, the mice need to be monitored at least twice a day, to examine in parallel their well-being (**steps 37–40**) and the electrical performance of the arrays (**steps 41–44**).

37. Ensure that the mice can move freely in the cage, that they have sufficient food and water, and that the bedding is dry and clean. Add food/water and/or replace bedding as needed.

***Note:*** Mouse handling (including feeding and water) should be done only by the researchers, and not by the animal facility staff. Handle the mice carefully to avoid damaging the array assembly.

***Note:*** Make sure the water bottles are not leaking, and if they are, recap them.

**Figure 7:**
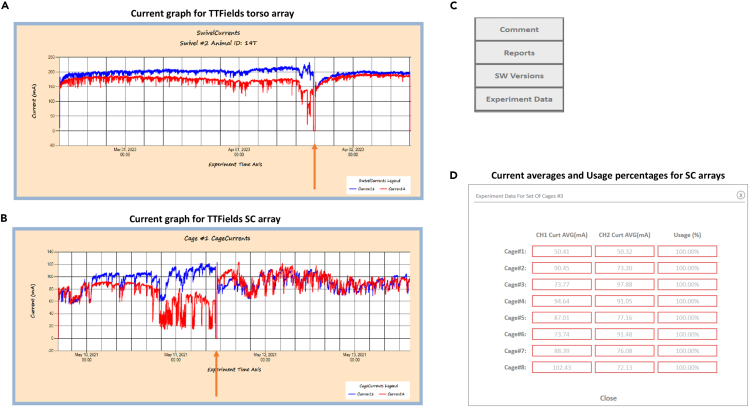
Reports Currents of channel 1 and channel 2 for a torso array, showing fluctuations and current decrease from the proper range, leading to treatment pause (orange arrow) and array replacement for improving the electrical performance (A). Currents of channel 1 and channel 2 for a SC array, showing fluctuations and current decrease from the proper range, leading to treatment pause (orange arrow) and array replacement for improving the electrical performance (B). Average currents and usage percentages for each cage connected to TTFields arrays may be seen via the Experimental Data button (C and D).

38. Weigh the mice every 2 days. Check for signs of apathetic or unresponsive behavior. If significant weight loss or other signs of bad health have been identified refer to troubleshooting 4.
  ***Note:*** The “with array” initial mouse weight you recorded following array assebly will serve as the baseline weight of the mouse
39. Check the mice’s skin for signs of damage or irritation along the sides of the adhesive tape. If such have been identified refer to troubleshooting 5.
40. SC array: Check the tumors for signs of abnormality or over-development. If such have been identified refer to troubleshooting 6.
41. Check that the PCB wires are straight and not coiled. If they are, refer to troubleshooting 2.
42. Check the electrical parameters for each mouse:
  a. Right-click on the screen background (Figure 2C) and press the Report button (Figure 7C) to open the current and the resistance graphs.
    ***Note:*** Current/resistance fluctuations over several hours, with decreasing currents or increasing resistance (see Table 1 for accepted values; Figures 7A and 7B for currents graphs) suggest poor array connectivity and deem array replacement. If such have been identified refer to troubleshooting 3.
  b. Right-click on the screen background (Figure 2C) and press the Experiment Data button (Figure 7C) to see for each cage the average current for each channel and the usage percentage (Figure 7D).
    ***Note:*** If an animal is presenting with usage of 85% or less, specific attention should be given to this animal so that the usage will not fall below 75% throughout the study, deeming exclusion from the study (as per the clinical recommendation for required usage).
43. Check the adherence and integrity of arrays and adhesive strips (for mice that did not require replacement of arrays in the previous stage due to problems with the electrical parameters). If it is compromised:
  a. Pause the treatment for the mouse requiring array arrangement by pressing the appropriate cage “Red Ear” button in the software (Figure 6F, indicated with a red arrow).
  b. Disconnect the array from the swivel, add additional strips to support the array or replace the strips if needed.
  c. Resume treatment by pressing the appropriate cage “Green Ear” button in the software (Figure 6F, indicated with a green arrow).
44. SC array: Check array cap intactness and replace it if needed.

***Note:*** Single array use should not exceed 4 days, perform an array replacement as needed. Maintain a uniform frequency of array replacements between the TTFields and heat groups.

### End your experiment

**Timing: 2–8 h (depending on the endpoint procedures and number of animals)**

Upon experiment end per protocol, the electric field application should be stopped, the arrays removed, and experimental endpoints and assays performed (per the study design).

45. Press the End Experiment button for each cage (Figure 6F, indicated with a blue arrow), then disconnect the array from the corresponding swivels.
46. After disconnecting all arrays, press the End Experiment (Figure 6F, indicated with a black arrow).
47. Make sure the desktop is selected as the location to save the experiment folder.
48. Verify that the following end-of-experiment signals appear: the two “turning wheels” stop, and the log production appears on the left part of the software display, followed by the final status turning to “ready”.

***Note:*** It takes some time for the log files to be generated and downloaded. You may in the meantime proceed to remove the arrays from the mice.

**Figure 8:**
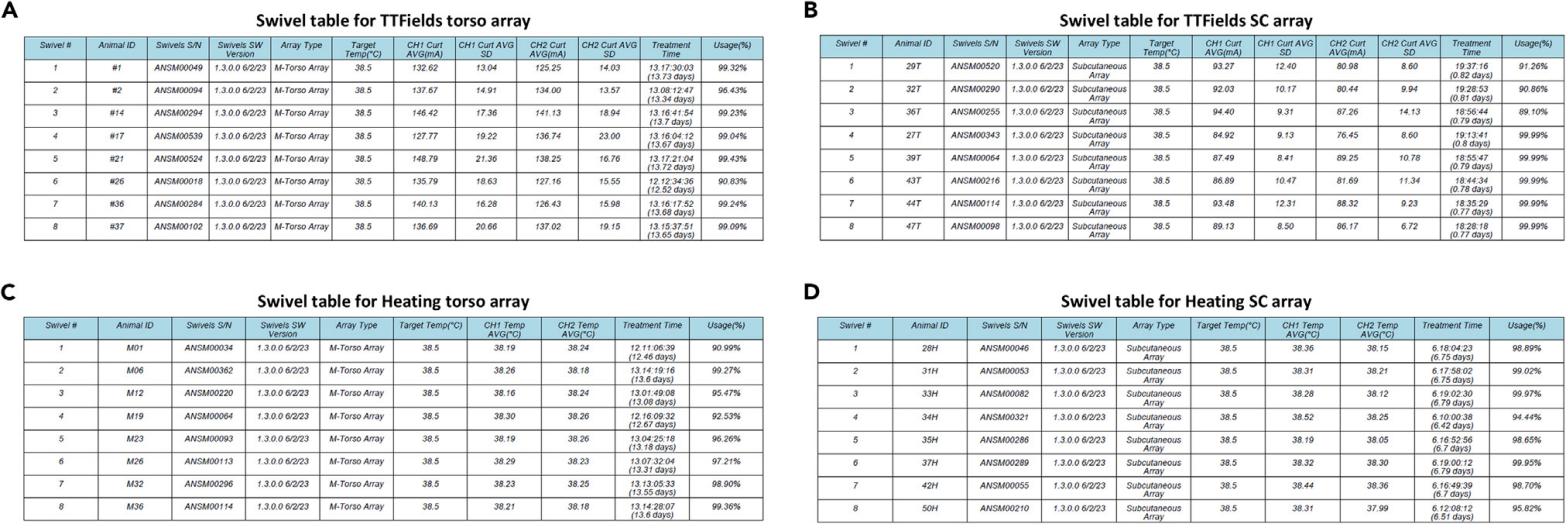
Swivel tables Swivel Table for treatment with TTFields torso arrays (A); TTFields SC arrays (B); heat torso arrays (C); and heat SC arrays (D).

49. Check that the experiment folder was saved and inspect the graphs and the Swivels Table (Figures 7A, 7B, and 8) in the Summary file to ensure data integrity.
50. Remove the arrays from the mice using an adhesive remover product (e.g., medical adhesive remover spray) and dispose of the arrays.
51. Perform end-point examinations planned by your study design, according to your standard protocols. This may include imaging, blood sampling, tumor collection, and more.

### Perform post-treatment maintenance of the inovivo system

**Timing: variable**

52. Remove the swivels from the cages, clean them with 70% alcohol wipes, and store them in a designated place.
53. Clean the generators with 70% alcohol wipes and store them in a designated place.
54. Remove the bedding and any remaining food from the cages and dispose of them according to your institutional guidelines. Wash the cages and all their parts (feeders, shelters, and bottles) with water and soap, making sure to remove any sticky residues and to leave them soap-free.
55. Autoclave the cages using a standard cycle of 20 min at 121°C. A drying step is recommended.
56. Store the cages properly to avoid dust accumulation.

## Expected outcomes

By following this protocol, you can examine the efficacy of TTFields for the inhibition of tumor development, using the mouse model of your choice and tumor type of your interest. You may also examine the effect of TTFields together with other cancer treatments. The expected level of tumor growth inhibition depends on the specific tumor examined and the duration of treatment. Make sure your control group is treated with sham heat arrays to account for animal stress and local heating by the TTFields treatment. Much attention needs to be given to animal handling and care for successful completion of the study, without needing to prematurely euthanize mice due to bad health conditions or retroactively exclude mice from the analysis due to insufficient TTFields delivery.

Attentiveness is needed to ensure the treatment is effectively applied, with adequate electrical currents and usage times throughout the study. To examine the treatment parameters, the system generated log files need to be inspected. A PDF file created at study termination named Summary presents the experimental information and calculated parameters. Specifically, the Swivels Table displays, per mouse, the average current for each channel (for the TTFields group, Figures 8A and 8B), the average temperature for each channel (for the heating group, Figures 8C and 8D), treatment time (Figures 8A–8D), and percent usage (Figures 8A–8D). Consider excluding from the final analyses TTFields-treated mice with average currents outside the desired values range (Table 1) and/or with usage lower than 75%.

Field measurements were performed (as described by Blatt et al.^8^) to verify field intensities were sufficient, above 1 V/cm.^9^ For the torso orthotopic arrays attached to C57BL/6J mice an intensity of 1.93 ± 0.45 V/cm was measured in the lungs, and an intensity of 2.67 ± 0.30 V/cm was measured in the pancreas (for a current of 125 mA and an animal weight of about 24 g). For the SC arrays attached to BALB/C mice, an intensity of 2.33 ± 0.50 V/cm was measured in tumors consistent of breast 4T1 cells (for a current of 85 mA).

## Limitations

The basic experimental conditions of an inovivo experiment, in which the mice need to be housed individually (to prevent wire entanglements) and assembled continuously with arrays around their torsos (which may limit movement and cause skin irritation), induce stress on the animals. This stress may cause weight loss, that if excessive, would require study termination ahead of time for the affected mouse. Skin damage from the depilation process and the continued array placment may require study discontinuation ahead of time for the affected mouse. The inovivo cages, swivels, and arrays material and structure were designed to reduce stress and skin irritation, by allowing dyadic mouse interaction, providing better comfort, reducing movement restriction, and furnishing better skin compatability. Still, the research team needs to pay close attention to reducing animal stress and skin damage. By acclimating the animals to the cages before study initiation, maintaining a constant cage-mate, providing supportive treatment (against weight loss and skin irritation), minimizing animal handling as possible, and minimizing the number of personnel handling the mice, the research team may contribute to successful completion of the study for the maximal number of animals.

Array replacement will occur several times for each mouse during an inovivo study, requiring anesthesia with isoflurane. Measures should be taken to minimize possible undesirable effects that repeated isoflurane inhalation may have on the mice. First, array status and function should be carefully and accurately assessed to avoid unnecessary replacements. Secondly, all required equipment should be prepared prior to anesthetizing the mice to minimize the duration under anesthesia. Ideally, skilled personnel should carry out the array replacements to further reduce anesthesia time. Finally, using isoflurane at the lowest effective concentration and providing thermal support will aid mouse recovery and reduce the risk of hypothermia, organ damage, and death.

Room environmental conditions should be monitored and strictly maintained within the proper range throughout the inovivo experiment – temperature of 18°C–20°C (below the IUCAC recommendation of 20°C–24°C due to heat generation by the treatment) and humidity of 40%–60%. High room temperature and high humidity may damage the chemical composition of the conductive material between the array and the animal skin, compromising TTFields delivery. Additionally, the discomfort for the mice from the arrays assembled around their bodies may be elevated under high room temperature and high humidity, possibly leading to increased stress, weight loss, and skin irritation.

Of note, the inovivo cages are not specific pathogen-free (SPF) cages. Therefore, treatment of immunodeficient mice with the inovivo system may be challenging. However, specific measures may be taken to overcome this caveat and allow such experiments: (1) wearing masks and gloves when handling the mice; (2) disinfection of gloves and work surfaces; (3) sterilization/disinfection of animal food, water, and bedding; (4) frequent cage replacement with clean autoclaved cages; (5) limiting the number of staff working with the mice; (6) minimizing the time from array package opening to array application; and (7) applying the disinfection agent to the mouse skin (steps 15–16) for a longer period of time.

## Troubleshooting

### Problem 1

A system error, detected by: illumination of the red ALARM LED of the generators, the port number not displayed for the swivels, or incorrect identification of cage status by the software, showing *No Cage* instead of *No Electrode* (set up the inovivo system; set experimental conditions on the inovivo system, **step 3**).

### Potential solution

Turn off the generator, then turn it on again. Disconnect the communication cables from the swivels and generators, then reconnect them. If a specific swivel sends an error, it may be faulty and needs to be replaced.

### Problem 2

An array is entangled, a swivel is showing a red ERROR LED, the swivels’ display does not show the correct array type, or the software is displaying in the Cages Data & Control Layout a note “Therapy not active”/”Heat not active” for any of the cages (connect the mice to the inovivo system and start the experiment, **steps 34** and **35**; monitor mice and arrays throughout the experiment, **step 41**).

### Potential solution

• If treatment is active, press in the software the respective cage “Red Ear” button to pause the treatment.
• Disconnect the array from the swivel, straighten the PCB wire, and then re-connect the array to the swivel.
• Press in the software the respective cage “Green Ear” button to resume treatment.
• If the problem is not resolved, press in the software the respective cage “Red Ear” button to pause the treatment.
• Disconnect the array from the swivel, replace the swivel, and then re-connect the array to the swivel.
• Press in the software the respective cage “Green Ear” button to resume treatment.
• If the problem is not resolved, refer to troubleshooting 3.

### Problem 3

Inadequate electrical performance (connect the mice to the inovivo system and start the experiment, **step 36**; monitor mice and arrays throughout the experiment, **step 42**) or a system error that was not resolved via troubleshooting 2.

### Potential solution

• Press in the software the respective cage “Red Ear” button to pause the treatment.
• Disconnect the array from the swivel.
• Anesthetize the mouse with isoflurane and remove the array using an adhesive remover product.
• Assemble a new array and reconnect the mouse to the system (assemble the arrays to the mice torsos, **steps 21–31**).

***Note:*** Before replacing the array, consider the wellbeing of the animal (monitor mice and arrays throughout the experiment, **steps 37–40**) and the time passed from the last array replacement to ensure the procedure will not impose unbearable stress on the animals. It is generally recommended to give 2–3 days between array replacements. If sufficient time has not passed, to avoid array replacement try adding or replacing the adhesive strips. If this does not resolve the problem and the mouse is in good health, replace the array even if only a short time has passed from the last replacement.

***Note:*** Before placing a new array, check for signs of skin damage or irritations. If such have been detected refer to troubleshooting 5.

***Note:*** Before placing a new array, remove any regrown fur by trimming and/or depilating as needed (prepare the mice by fur removal from the torsos, **steps 6–20**).

**CRITICAL:** Mice should always be returned to their original cage with their original cage-mate.

• Press in the software the respective cage “Green Ear” button to resume treatment.

### Problem 4

A mouse shows signs of poor health, such as weight loss or apathetic/unresponsive behavior and indications of poor health (monitor mice and arrays throughout the experiment, **step 38**).

### Potential solution

• Press in the software the respective cage “Red Ear” button to pause the treatment.
• Disconnect the array from the swivel.
• Anesthetize the mouse with isoflurane and remove the array using an adhesive remover product.
• Weigh the mouse and compare it with mouse’s initial weight. If weight loss is detected, administer Ringer’s lactate solution or similar to the subcutis, consider changing or adding drugs to the analgesic protocol, and consider letting the mouse rest from treatment. Consult with the attending veterinarian.

**CRITICAL:** For cases of severe weight loss treat the mouse according to your regional ethics requirements.

***Note:*** Check for signs of skin damage or irritations. If such have been detected refer to troubleshooting 5.

• Assemble a new array and reconnect the mouse to the system (assemble the arrays to the mice torsos, **steps 21–31**).

***Note:*** Before placing a new array, check for signs of skin damage or irritations. If such have been detected refer to troubleshooting 5.

***Note:*** Before placing a new array, remove any regrown fur by trimming and/or depilating as needed (prepare the mice by fur removal from the torsos, **steps 6–20**).

**CRITICAL:** Mice should always be returned to their original cage with their original cage-mate.

• Press in the software the respective cage “Green Ear” button to resume treatment.

### Problem 5

A mouse shows signs of skin damage or irritations (monitor mice and arrays throughout the experiment, **step 38**).

### Potential solution

• Press in the software the respective cage “Red Ear” button to pause the treatment.
• Disconnect the array from the swivel.
• Anesthetize the mouse with isoflurane and remove the array using an adhesive remover product.
• Apply a topical agent to the mouse skin according to your veterinarian’s instructions for treating the specific skin condition. Consult the veterinarian regarding amending the analgesic protocol with respect to the skin condition.
• Return the mouse to the cage and let it rest from the treatment.

**CRITICAL:** Mice should always be returned to their original cage with their original cage-mate.

• Clean any residues of the topic product from the skin.
• Assemble a new array and reconnect the mouse to the system (assemble the arrays to the mice torsos, **steps 21–31**).

***Note:*** Before placing a new array, remove any regrown fur by trimming and/or depilating as needed (prepare the mice by fur removal from the torsos, **steps 6–20**).

**CRITICAL:** Mice should always be returned to their original cage with their original cage-mate.

• Press in the software the respective cage “Green Ear” button to resume treatment.

### Problem 6

For SC arrays, tumors are showing signs of abnormality or over-development (monitor mice and arrays throughout the experiment, **step 38**).

### Potential solution

• Press in the software the respective cage “Red Ear” button to pause the treatment.
• Disconnect the array from the swivel.
• Anesthetize the mouse with isoflurane and remove the cap from the tumor.
• Measure the tumor using a Vernier caliper. If at least one of the tumor dimensions exceeds 10 mm the tumor will no longer fit within the array opening. Terminate the experiment for the specific mouse.
• If necrosis or ulcerous have been identified:
  ○ Remove the array using an adhesive remover product.
  ○ Apply a topical agent to the mouse skin according to your veterinarian’s instructions for treating the specific skin condition.
  ○ Return the mouse to the cage and let it rest from treatment until showing improvement (up to 24 h).
    **CRITICAL:** Mice should always be returned to their original cage with their original cage-mate.
  ○ Clean any residues of the topic product from the skin.
  ○ Assemble a new array and reconnect the mouse to the system (assemble the arrays to the mice torsos, **steps 21–31**).
    ***Note:*** Before placing a new array, remove any regrown fur by trimming and/or depilating as needed (prepare the mice by fur removal from the torsos, **steps 6–20**).
    **CRITICAL:** Mice should always be returned to their original cage with their original cage-mate.
  ○ Press in the software the respective cage “Green Ear” button to resume treatment.

## Resource availability

### Lead contact

Further information and requests for resources and reagents should be directed to and will be fulfilled by the lead contact, Moshe Giladi, (mosheg@novocure.com).

### Technical contact

Technical questions on executing this protocol should be directed to and will be answered by the technical contact, Sewar Zbidat (szbidat@novocure.com).

### Materials availability

This study did not generate new unique reagents.

### Data and code availability

This paper does not generate any dataset or code.

## Acknowledgments

The study was conducted and funded in collaboration with Novocure Ltd. (Haifa, Israel), with additional funding sources and support from the Minnesota Ovarian Cancer Alliance Ovarian Cancer Research Award (PI: E.L.), the American Cancer Society Research Scholar Grant (RSG-22-022-01-CDP, PI: E.L.), and the 2019 American Association for Cancer Research (AACR)-Novocure Tumor Treating Fields Research Grant (grant number 1-60-62-LOU; PI: E.L.). We acknowledge and thank members of the Research Animal Resources (RAR) core facility staff at the University of Minnesota for assistance on this project. We would also like to thank Chelsea Higgins, PhD, of Novocure for editorial assistance and Meshi Levi of Novocure for illustration development.

## Author contributions

S.Z., S.D., R.B., S.C., Y.P., and M. Giladi designed the protocol. T.J.G., K.D., S.P., K.L., A.S., X.Z., S.S., and E.L. conducted and corrected the protocol. M. Gabay performed field intensity measurements. S.Z. and M.S. coordinated protocol steps photography. T.J.G., K.D., S.Z., Y.P., A.H., S.S., and E.L. drafted the manuscript with input from all other authors. All authors reviewed and approved the manuscript.

## Declaration of interests

E.L. reports honoraria and travel expenses for lab-based research talks, 2018–2021, and equipment for laboratory-based research, 2018–present, from Novocure, Ltd. (Haifa, Israel); honorarium for a panel discussion organized by Antidote Education for a CME module on diagnostics and treatment of HER2+ gastric and colorectal cancers, funded by Daiichi Sankyo, 2021 (honorarium donated to lab); and compensation for scientific review of proposed printed content from Elsevier Publishing and Johns Hopkins Press. E.L. is a consultant for NomoCan Pharmaceuticals (no financial compensation); a Scientific Advisory Board Member of Minnetronix, LLC, 2018–2019 (no financial compensation); a consultant and speaker honorarium for Boston Scientific US, 2019; and a shareholder of Ryght, Inc. E.L. is an Institutional Principal Investigator for clinical trials sponsored by Celgene, Novocure, Intima Bioscience, and the National Cancer Institute and has University of Minnesota membership in the Caris Life Sciences Precision Oncology Alliance (no financial compensation). S.Z., S.D., R.B., S.C., M. Gabay, M.S., I.T., Y.P., A.H., and M. Giladi are employees of Novocure and hold company stocks and TTFields-related patents.
